# Clinical course, characteristics and prognostic indicators in patients presenting with back and leg pain in primary care. The ATLAS study protocol

**DOI:** 10.1186/1471-2474-13-4

**Published:** 2012-01-20

**Authors:** Kika Konstantinou, Ruth Beardmore, Kate M Dunn, Martyn Lewis, Samantha L Hider, Tom Sanders, Sue Jowett, Simon Somerville, Siobhan Stynes, Danielle AWM van der Windt, Steven Vogel, Elaine M Hay

**Affiliations:** 1 Arthritis Research UK Primary Care Centre, Primary Care Sciences, Keele University, Staffordshire ST5 5BG, UK

## Abstract

**Background:**

Low-back related leg pain with or without nerve root involvement is associated with a poor prognosis compared to low back pain (LBP) alone. Compared to the literature investigating prognostic indicators of outcome for LBP, there is limited evidence on prognostic factors for low back-related leg pain including the group with nerve root pain. This 1 year prospective consultation-based observational cohort study will describe the clinical, imaging, demographic characteristics and health economic outcomes for the whole cohort, will investigate differences and identify prognostic indicators of outcome (i.e. change in disability at 12 months), for the whole cohort and, separately, for those classified with and without nerve root pain. In addition, nested qualitative studies will provide insights on the clinical consultation and the impact of diagnosis and treatment on patients' symptom management and illness trajectory.

**Methods:**

Adults aged 18 years and over consulting their General Practitioner (GP) with LBP and radiating leg pain of any duration at (n = 500) GP practices in North Staffordshire and Stoke-on-Trent, UK will be invited to participate. All participants will receive a standardised assessment at the clinic by a study physiotherapist and will be classified according to the clinically determined presence or absence of nerve root pain/involvement. All will undergo a lumbar spine MRI scan. All participants will be managed according to their clinical need. The study outcomes will be measured at 4 and 12 months using postal self-complete questionnaires. Data will also be collected each month using brief postal questionnaires to enable detailed description of the course of low back and leg pain over time. Clinical observations and patient interviews will be used for the qualitative aspects of the study.

**Discussion:**

This prospective clinical observational cohort will combine self-reported data, comprehensive clinical and MRI assessment, together with qualitative enquiries, to describe the course, health care usage, patients' experiences and prognostic indicators in an adult population presenting in primary care with LBP and leg pain with or without nerve root involvement.

## Background

Low back pain (LBP) is common, affecting 70-80% of the population at some point in their lives [1]. The presence of pain radiating down the leg is associated with a poor prognosis in patients with LBP [2-5]. Patients who complain of low back and leg pain suffer more severe pain and disability, take longer to recover and lose more time from work [6-12]. Lumbar spinal radiculopathy or nerve root pain represents one distinct presentation of low back-related leg pain, which is generally characterised by pain radiating to below the knee and into the foot and toes. Although the prevalence of nerve root pain is much less than that of LBP alone [13], the condition is considered responsible for most of the indirect costs and lost workdays associated with back pain [14,15].

Compared to the literature investigating prognostic indicators of outcome for LBP, there is limited evidence on prognostic factors for low back-related leg pain including the group with nerve root pain [16]. It is unclear at present whether the prognostic indicators relevant to outcome in patients with leg pain are similar to those for LBP alone with published literature providing conflicting views at times [17,18].

Recent LBP research has moved away from a "one-size fits all" approach, where heterogeneous groups of patients receive broadly similar treatments, to studies which address the question of 'who does better with what'. This new approach investigates whether outcome is better when subgroups of LBP patients identified according to their prognostic profile or to specific characteristics, are matched with appropriate treatments. Preliminary findings support the view that different categories of 'symptoms and signs' in LBP patients can be identified, which seem amenable to specific treatments [19-22]. Our own research shows that stratifying patients with non-specific low back pain (including those with leg pain) according to their risk of poor outcome (i.e. prolonged disability) and providing treatments matched each strata, results in better clinical and cost outcomes compared to current best (non-stratified) care [23]. This line of enquiry, although intuitively applicable to the more specific presentation of leg pain occurring in association with LBP, has not been evaluated, especially in primary care where most patients present and are managed. There is a need therefore for studies exploring prognostic indicators of outcome in the subgroup of patients with low back and leg pain in order to inform future research on future stratified management.

Clinical characteristics and indexes of risk however, are not the only variables affecting outcome. The effect of interaction style (level of patient participation, engagement of the clinician with patient concerns, elicitation of patient preferences, and reassurance) on patient health outcomes has received limited attention in the literature. However, research has shown that the specific approach adopted by clinicians in the consultation may lead to significant improvements in health outcomes [24,25]. Consequently, there is a need to investigate the different dimensions of the physiotherapist-patient interaction in musculoskeletal care, to identify the relationship between consultation structure, and subsequent outcomes (e.g. concordance to clinical advice). Furthermore, previous research has not specifically investigated the effect of leg pain, and its diagnosis and management, on patients' experiences in primary care.

### Aims and objectives

(i) The primary aims of this study are: for patients presenting in primary care with low back and leg pain, managed according to currently agreed "best practice" and followed up for 12 months, to describe the clinical, imaging, demographic characteristics and health economic outcomes for the whole cohort, and to investigate differences between those classified with and without nerve root pain

and

(ii) to identify prognostic indicators of outcome (i.e. change in disability at 12 months), for the whole cohort and, separately for those classified with and without nerve root pain

Specific objectives include:

(a) identification of prognostic subgroups of patients with distinct trajectories of pain and disability over 12 month follow-up

(b) description of health care resource use and work productivity within and between those with LBP and non-specific leg pain, and those with a clinical diagnosis of LBP with nerve root pain over 12 months follow-up

(c) investigation of the effect of clinical assessment and treatment negotiation between physiotherapists and patients on decision-making

(d) examination of whether the classification of leg pain (eg. either with or without nerve root involvement) impacts on patients' symptom management, help seeking behaviour and illness trajectory

## Methods/design

Ethical Approval for this study has been granted by the South Birmingham Research Ethics Committee (REC ref. 10/H1207/82).

### Study design and setting

Primary care, multi-centre prospective observational cohort study

### Participants

Adults aged 18 years and over consulting their General Practitioner (GP) with LBP and radiating leg pain of any duration at GP practices in North Staffordshire and Stoke-on-Trent, UK. Radiating leg pain is defined as pain spreading from the back beyond the gluteal fold to anywhere in the leg. In this context "pain" is taken to include all unpleasant/abnormal sensations such as 'pins and needles' or numbness.

### Exclusion criteria

• Persons with "red flags" indicative of possible serious spinal pathology

• Serious co-morbidity which prevents patients from being able to travel to the clinic and/or undergo a clinical assessment

• Patients with serious mental health problems who the GP considers to be vulnerable and for whom participation in the study would be detrimental

• Previous spinal surgery

• Pregnancy

• Currently receiving physiotherapy (or osteopathy, chiropractic) or under a secondary care consultant for the same problem

• Not able to read and speak English

### Recruitment procedure

Potentially eligible participants will be identified at the GP consultation and referred to a Community Low Back and Leg Pain Clinic where they will be invited to take part in the study. When a patient with back pain consults their GP, and the GP enters an appropriate Read Code on the computer system, a "pop-up" prompt screen will ask the GP if they think the patient has leg pain associated with their back problem, and if so to consider whether the patient is suitable to be invited to the Community Low Back and Leg Pain Clinic. The GP will have the facility to enter "yes" or "no" on the computer system to flag if patients are suitable or unsuitable for referral to the clinic. Whilst desirable, it is not imperative that the GP informs the patient about the clinic for example, if the GP enters the patient as suitable on the system but after the consultation has ended and the patient has left, the patient will still be invited through the process described below.

On a weekly basis West Midlands North Primary Care Research Network (WMN PCRN) staff will facilitate the mailing of letters to all patients who have been flagged as suitable. The letter will invite patients to telephone the clinic administrator to make an appointment at the clinic, it will explain that there is a research study being hosted at the clinic but that attendance at the clinic does not oblige them to take part. Patients who telephone the clinic administrator will be offered a clinic appointment within 10 working days. A letter will be sent to patients to confirm their appointment time--a study baseline questionnaire and patient information sheet will be enclosed with this letter. Approximately 2 days before the clinic, a clinic nurse will telephone patients to remind them about their appointment time and ask those who are interested in taking part in the research to bring their completed questionnaire.

There will also be a separate referral pathway to capture patients who contact PhysioDirect (Physiotherapy Direct Access Service), a community physiotherapy service allowing patients to telephone directly through to a physiotherapist without needing to first consult their GP. Following the usual PhysioDirect assessment, suitable patients will be asked whether they would be interested in being invited to the Community Low Back and Leg Pain Clinic. Referrals will be faxed to the research centre and a letter will be sent to the patient inviting them to telephone the clinic administrator to make an appointment for the clinic as described above.

See Additional file 1 for a flowchart of the study.

### Community low back and leg pain clinic

Physiotherapy assessment and management will be provided to all patients attending, irrespective of whether or not they are eligible or agree to take part in the study. The clinic will be operated as an integrated service/research clinic, with NHS treating clinicians being fully supported by WMN PCRN clinical and administrative staff working as a single team. At the clinic, a nurse will check potential eligibility and if the patient wishes to take part in the study gain signed informed consent. The study questionnaire which had been mailed to patients with their appointment letter will be received and checked and participants will be asked to complete a further brief baseline questionnaire during the clinic.

All study participants will receive a standardised assessment at the clinic by a study physiotherapist, at which full eligibility will be established by, for example, by excluding patients whose leg pain is not considered to be associated with their back pain (e.g. hip osteoarthritis). This standardised assessment has been developed following a Delphi study aimed at developing consensus on the content of the clinical assessment for adults presenting in primary care with low back-related leg pain [26]. Each eligible patient will be classified by the assessing physiotherapist as having LBP with referred leg pain or having LBP with nerve root pain ("reference standard" is the clinician's diagnosis). The content of the clinical assessment is provided in Additional file 2.

### Care pathways

Clinical management will follow agreed care pathways, based on current best clinical evidence, practice guidelines and local services and resources. In addition to clinical examination findings simple self-reported clinical markers of severity and risk of poor outcome measured using the STarT Back tool [27], will be used as an adjunct to clinical judgment to direct patients' care. The patient's care pathway will be decided at the discretion of the treating clinician in consultation with the patient and in line with the agreed care pathways. This is a mixed stratified/stepped care approach and as such if a patient's symptoms deteriorate they can transfer to a pathway for patients with more severe symptoms. There will be three categories/groups according to symptoms severity and previous response to treatment for current episode.

### Group (i)

Patients in this group are expected to have mild/improving symptoms and be classified at low risk of a poor outcome according to the STarT Back tool. Patients will receive a brief physiotherapy intervention (one or two sessions) according to their needs consisting of advice, education and home exercises. The physiotherapist will emphasise messages about promoting speedy return to normal activity, avoiding rest, appropriate use of pain relieving modalities (such as painkillers), and work issues. Patients will be encouraged to ask questions relating to any specific concerns about their back and leg pain. As appropriate to their presentation, patients will be given some written educational information--if it is suitable this may be the 'Back Book' [28], or patients with nerve root pain may be given more specific information relating to their condition. If appropriate/necessary patients will be given an information sheet containing local contacts for exercise venues such as swimming pools and gyms, exercise on prescription and self-help groups to facilitate activity and early return to work. Patients will have the option to contact the service again if symptoms persist or worsen.

### Group (ii)

Patients in this group are expected to have moderately severe pain and disability and most likely be at "moderate" or "high" risk of poor outcome according to the STarT Back tool. For those patients classified as 'moderate risk' of a poor outcome, the physiotherapist will negotiate an individualised treatment plan with the patient according to their need and best current evidence. The physiotherapist will address any worries or fears and unhelpful beliefs patients' may have about their back and leg pain, will emphasise messages as for group (i), and will use a range of pre-agreed physiotherapy techniques, including manual therapy and exercises where appropriate. For those patients classified as 'high risk' of a poor outcome, in addition to appropriate treatments for their back-related physical symptoms (e.g. pain), treatment may also include cognitive behavioural approaches specifically addressing the main psychosocial risk factors for chronicity (pain related fear about movement, catastrophising and pain-related depression).

The group (ii) intervention will be delivered in one 45-min session with a target of up to 6 further 30-min sessions (tailored according to clinical need) over 6-8 weeks. Pathways will be in place to refer patients that fail to improve or worsen to the dedicated Back Pain Service, for further assessment and management. The Back Pain Service is a pathway run by spinal physiotherapy specialists and there is access to and input from orthopaedic surgeons, pain clinic anaesthetists and rheumatologists as appropriate. Management of patients referred to the Back Pain Service will be at the discretion of the treating specialist, but may involve invasive procedures such as injections or surgery where indicated.

### Group (iii)

Patients in this group will have severe levels of back and leg pain-related symptoms with associated functional limitations, and/or progressive neurological symptoms or signs, with or without significant levels of psychological distress (they are expected to be classified as "moderate" or "high" risk of poor outcome according to the STarT Back tool), and are thus considered not appropriate for conservative primary care physiotherapy interventions and/or cognitive behavioural approaches. In addition Group (iii) may include patients in whom such treatments have been tried previously without any benefit. After the initial clinic assessment these patients will be referred directly to the dedicated Back Pain Service as described above.

### Training of physiotherapists

Seven senior, experienced musculoskeletal physiotherapists will undertake 21/2 days training led by the study Principal Investigator (KK) and including sessions with a GP with special interest in musculoskeletal conditions (GPwSI MSK), a Rheumatologist, and a Consultant in Pain Management. The focus of the training is on carrying out the standardised assessment according to agreed protocols, and on equipping the physiotherapists to assess and target back pain, leg pain, co-morbid pain, disability and psychological risk factors for chronicity such as pain related distress, fear of movement, unhelpful beliefs and expectations. Emphasis is given on the differentiation between back pain with leg pain due to nerve root involvement and back pain with referred leg pain. The training includes the evidence-based assessment of LBP, including the use and interpretation of the STarT Back tool to guide treatment in addition to the role of diagnostic investigations (including understanding, interpretation and communication of MRI results to the patients and their GPs), medication, epidural injections and surgery in back pain and radiculopathy. Current guidelines for managing LBP in primary care will be discussed, including appropriate reassurance and advice about analgesia, the maintenance of, or return to, usual activities (including work) and patients who present a clinical or management concern (e.g. those with signs of potential serious pathology or red flags). The training includes current best physiotherapy practice for the management of disability, back pain and leg pain, including the role of exercise and manual therapy as well as strategies for equipping patients with the skills to manage future recurrences. Goal setting, pacing and graded exercise will also be covered.

The training will be supplemented by a comprehensive manual, providing clear guidelines and treatment algorithms for the evidence based assessment and treatment of patients with LBP, either with or without leg pain. Patients exhibiting many psychosocial risk factors for chronicity may be treated, if necessary, by physiotherapists that have already undergone extensive appropriate training in the context of our previous STarT Back trial [23,29] and implementation study [30]. Continuous mentoring and supervision for the duration of the study will be provided by the Principal Investigator and the spinal physiotherapy specialists working in the Back Pain Service.

### MRI scans

All participants will be invited for a Magnetic Resonance Imaging (MRI) scan, providing there are no contraindications, unless they have had an MRI scan in the past 6 months and their clinical presentation has not changed. MRI is the best available diagnostic imaging modality for LBP and leg pain as it provides excellent resolution of both nerve roots (allowing for assessment of nerve root compression) and bony structures. MRI is non-invasive for the patient and does not require any ionising radiation exposure. MRI will be performed using 1.5T magnetic resonance units. A body spine surface coil will used for imaging the lumbar region. Patients will undergo a standard lumbar spine MRI (including sagittal T1 and T2 weighted spin echo sequences and STIR sequences), similar to that undertaken in routine clinical practice. This will take approximately 30 min with the patient lying in the supine position in the scanner.

A summary report on the MRI scan will be provided by a Consultant Radiologist at the NHS Trust Hospital. The primary purpose for the MRI scan is to provide data for the research study (as opposed related to clinical need) and will be completed within 10 working days of the clinic appointment. However, any relevant clinical findings from the scan will be acted upon by the treating clinician. The patients and their GPs will be notified of the results as a matter of course.

### Qualitative data collection

Qualitative research methods will be used to address the study's specific objectives (c) and (d). This will involve a series of observations (approximately 30) during the standardised clinical assessments and subsequent treatment consultations, as well as interviews with a sample of patients (approximately 20). Consultations for observation and participants for interview will be selected 'opportunistically' from each of the three care pathways ensuring that a wide range of patients (in relation to age, sex, symptom severity, and social class) are included ('maximum diversification sample'). Observations will be conducted in person by a qualitative researcher using a topic guide in order to gain experiential insights and understanding of the consultation, the participants, and wider organisational influences. Researcher field notes will be supplemented with audio-recordings where participants have given consent.

Observations will empirically examine the treatment decision-making process, the involvement of patients in these decisions, and the role of symptom severity/duration on 'process' issues. We hypothesise that these issues could have a bearing on future patient outcomes, which will be examined in more detail during the patient interviews. The consultations will compare patients with and without a clinical diagnosis of 'nerve root involvement'. Observations will examine whether diagnostic status appears to influence the clinical decision making process of physiotherapists, and choices of treatment expressed by patients at different stages of treatment within each care pathway. Hence, observations will be conducted at various stages of treatment in order to explore whether the physiotherapist-patient interaction changes over time and the potential impact on patient 'concordance to the treatment intervention.

Patients will be invited to take part in the interview study following their consultation with a physiotherapist. Semi-structured qualitative interviews will be conducted using a topic guide to explore key themes systematically with all participants, but with emergent observations and insights from earlier findings feeding into subsequent interviews. This will allow examination of variation in perceptions between participants. Patients will be interviewed to explore: 1) patients' experiences of, and perceptions towards, decision-making with a physiotherapist, and 2) patients' illness trajectories and acceptability of the treatment plan. Interviews will specifically examine how patients' 'diagnostic status' (whether they receive a diagnosis of 'nerve root involvement' or not) affects their illness experience, pain management and help seeking behaviour.

### Quantitative data collection

The study outcomes will be collected at 4 and 12 months using postal self-complete questionnaires. Data will also be collected each month using brief postal questionnaires to enable detailed description of the course of low back and leg pain. Reminders will be sent to non-responders at 4 and 12-months.

The primary outcome for this study is disability at 12 months follow up as measured with the Roland and Morris Disability Questionnaire (RMDQ) leg pain version [31,32]. Secondary outcomes include measures of pain intensity and trajectory, neuropathic pain, psychological constructs, work-related factors, general health perceptions and health care utilisation. Measures of disability, pain intensity, and work absence will be collected in the brief monthly questionnaires in order to describe the detailed course of the condition. Additional potential prognostic factors collected at baseline include, episode duration, pain location, clinical assessment findings (i.e. neurological examination findings), co-morbidity, smoking, height and weight (see Table 1 for details of measures and time points for data collection).

**Table 1:** Outcome Measures

Domain	Measure	Baseline	4 Month	12 Month
Disability*	Roland and Morris Disability Questionnaire (RMDQ) leg pain version (Roland and Morris 1983 [31]; Patrick et al. 1995 [32])	Yes	Yes	Yes

Pain Intensity*	Current, average and 'least' pain in last 2 weeks for both back and leg pain--numerical rating scales (Dunn et al. 2010 [38])	Yes	Yes	Yes

Employment*	Questions on employment status, work absence, sick certification and 'struggling at work'	Yes	Yes	Yes

Risk of poor outcome	STarT Back Tool (Hill et al. 2008 [27])	Yes	Yes	Yes

Leg Pain	Above/below the knee; and Sciatica Bothersomeness Index (SBI) (Grovle et al. 2010 [39])	Yes	Yes	Yes

Episode Duration	Current episode for each back and leg pain, plus time since 'pain free month' (Dunn and Croft 2006 [40])	Yes	_	_

Pain Location	Pain Manikin (Lacey et al. 2005 [41])	Yes	_	_

Illness Perceptions	Musculoskeletal Illness Perceptions Questionnaire (IPQ-R) Short-Form (adapted from Moss-Morris et al. 2002 [42])	Yes	Yes	Yes

Self-Efficacy	Pain Self-Efficacy Questionnaire (PSEQ) (Nicholas 2007 [43])	Yes	Yes	Yes

General Health	SF-1 (Ware 2000); and EQ5D (EuroQol Group 1990 [44])	Yes	Yes	Yes

Change	Global Assessment of Change--single question	No	Yes	Yes

Productivity	Performance at work--single numerical rating scale	Yes	Yes	Yes

Health Care Utilisation	Health Care Utilisation Questions	No	Yes	Yes

Neuropathic Pain	S-LANSS (Bennett et al. 2005 [45])	Yes	Yes	Yes

Work Load	Measure of physical work load (adapted from Miranda et al. 2002 [10])	Yes	_	_

Co-morbidity	Co-morbid health conditions & symptoms	Yes	_	_

Pain Trajectory	Single question (based on Dunn et al. 2006 [40])	Yes	_	Yes

Anxiety and Depression	Hospital Anxiety and Depression Scale (HADS) (Zigmond and Snaith 1983 [46])	Yes	_	Yes

Satisfaction with Care	Single question	_	Yes	_

Smoking	Self-report questions	Yes	_	_

Height/Weight	Measured at Clinic Assessment	Yes	_	_

*Disability, pain and work absence will be collected monthly (brief questionnaires will be used on months when full outcomes are not being collected)

### Health economics

The study includes a health economic evaluation focusing both on a health care perspective and the wider societal perspective. A health care perspective addresses the direct health care costs incurred within both the public and private sectors. The societal perspective will incorporate the indirect costs of sickness absence and reduced productivity at work due to low back and/or leg pain. Health care use data will focus on key cost drivers including hospital attendances, surgery, inpatient stays, outpatient appointments and any other hospital visits to health care practitioners within the UK National Health Service (NHS) and private practice, consultations with NHS primary health care providers (e.g. general practitioner, practice nurse), prescribed medications, and over-the-counter treatments. This data will also capture resource use required to implement the care pathway. Unit costs assigned to these resources will be obtained from standard published sources reflecting UK national averages.

### Sample size

A recruited sample size of 500 patients will be sufficient to detect, with at least 80% power, a difference of 15% in the proportion of people with poor outcome, in relation to back-related disability, between the subgroups of leg pain patients with and without nerve root pain, assuming 20% loss to follow up and 5% two-tailed significance level. Poor outcome is defined as less than 30% change on RMDQ score [33]. This sample size is likely to be adequate for exploring the independent association of at least 15 prognostic indicators of outcome per subgroup (according to Altman's [34] guideline for restriction of the number of variables in a multiple regression). Based on findings from our previous observational and intervention studies of back pain we can assume a 6% prevalence of adults consulting their GP with LBP, of which about 60% will report leg pain, and of these the GP will invite approximately two-thirds to the community clinic. Of those invited we anticipate about 50% will attend, of which about two-thirds will be interested in taking part in the research study. We expect two-thirds of those who undergo the eligibility screening with the study nurse and physiotherapist will be suitable for the study. This means that approximately 1200 patients will need to be seen at the community clinic to recruit 500 participants to the cohort study, which requires a total GP practice population of about 90,000 adults, equivalent to about 12 average-sized practices in the WMN PCRN network.

### Analysis

#### Quantitative

Clinical, imaging and demographic characteristics, health economic and clinical outcomes of patients with back and leg pain will be described for the whole cohort and separately according to the presence/absence of nerve root pain. Estimates of mean differences (with 95% confidence intervals) in baseline and outcome measures across all endpoints will be evaluated between those with and without nerve root involvement. The mean absolute and change scores for the Roland and Morris Disability Questionnaire (RMDQ, primary outcome measure) will be analysed across all monthly time points.

As stated, the primary outcome is "improvement" in the RMDQ: defined as a change of at least 30% improvement in an individual's RMDQ score [33]. Direct statistical testing will be based on a comparison of outcomes between leg-pain patients with and without classified nerve root pain. Additional exploratory analyses will be carried out for example comparing patients allocated to each of the three care pathways. A difference of 15% in the proportion of patients who "improve"/"do not improve" according to the primary outcome between subgroups stratified according to the presence/absence of nerve root pain is deemed to be significant from a clinical perspective and for the purposes of statistical hypothesis testing.

Associations between baseline indicators (including self-report items and clinical assessment findings) and back-pain related disability at 4 and 12 months will be investigated to identify indicators of poor outcome. Potential prognostic factors will be investigated for the total sample, and separately for those classified by nerve-root pain.

Statistical modelling including linear and logistic regression methods will be carried out for numerical and categorical outcome measures, respectively, in order to investigate the association between potential prognostic factors and outcome. Inclusion of longitudinal terms within an appropriate repeated-measures framework will be adopted for the evaluation of the primary outcome measure. Random effects modelling and multiple imputation methods will be considered to address the limitations due to expected missing data during follow up assessments and to enhance the power of the statistical evaluation. Interaction terms (e.g. with time and clinical subgroups) will be included and linear/non-linear associations explored.

Longitudinal latent class analysis will be used to explore the course of the condition and identify clusters of patients with distinct trajectories of symptoms over 12 months follow up, making use of the monthly assessments of the primary outcome measure [35,36].

#### Health economics

The health economic analysis will concentrate on how costs and outcomes vary between sub-groups of patients, including diagnostic sub-groups and severity of symptoms at baseline. In addition, patterns of cost and outcome change over time will be explored. Multiple imputation will be used if there is missing cost and/or outcome data. Descriptive statistical analysis will be undertaken to describe the health care resource use, outcomes and employment status (loss in productivity) within and between sub-groups during the one-year follow-up period. Regression-based analyses will be used to explore the variation in key outcome variables, notably costs and health measures such as EQ-5D, and their associations with a range of possible explanatory variables.

#### Qualitative

The analysis of the observations and interviews will commence during fieldwork. Early findings and insights will inform subsequent qualitative data collection. These findings will in turn inform the development of theory from the data (grounded theory). Both the qualitative interview and observation data will be coded using the N-Vivo qualitative data analysis software. Each data set will be coded using a separate coding frame reflecting the nature of the data (perceptions and process). The main themes in the interview and observation data will be identified and analysed using the 'constant comparative' method [37]. The coded interview data will be compared systematically with the observation data to aid conceptual development and to identify connections between the two data sets. Observations will address process issues, or show empirically *how *decisions are negotiated during consultations, whilst interviews will examine perceptions, or *why *things happen. The analysis will help to identify the factors affecting treatment negotiations between physiotherapists and patients by exploring the inter-personal dynamics of consultations, alongside patients' experiences of low back and leg pain, adopting a prospective and retrospective design.

#### Project timeline

Recruitment to the study began in April 2011 and is due to be completed at the end of 2012. The 12-month follow-up (including reminders and time for response) should be finished early 2014, which will be followed by final data cleaning and analysis.

## Discussion

This study will provide information on the course and prognosis of pain, disability and other health outcomes in patients presenting with back and leg pain in primary care, assessed according to a standardised schedule for the presence or absence of nerve root pain and treated according to current best practice. The study will inform the approach to more effective targeting of treatment, which will be investigated in future trials. This proposed study will add to best practice for assessing and managing LBP with leg pain in the primary care setting, with the potential to improve patient outcomes and reduce costs to both society and the health care system. It may guide development of primary care services for back pain patients, locally and nationally. The qualitative observations will describe the content of the physiotherapist-patient interaction across the three care pathways, identify the possible effects on patient outcomes (eg. concordance with clinical advice), and suggest improvements for delivering care to patients and for informing physiotherapist training. The patient interviews will elicit patients' perceptions of the different care pathways, and examine how patients with, and without, a clinical diagnosis of 'nerve root involvement' respond to treatment.

## Competing interests

The authors declare that they have no competing interests.

## Authors' contributions

All authors participated in the design of the study and the drafting of the manuscript. All authors have read and approved the final manuscript.

## Pre-publication history

The pre-publication history for this paper can be accessed here:

http://www.biomedcentral.com/1471-2474/13/4/prepub

## Supplementary Material

Additional file 1 **Study Flowchart**. Click here for file

Additional file 2 **Clinical Assessment Form**. Click here for file

## References

[B1] van Tulder M Koes B Bombardier C Low back pain Best Pract Res Clin Rheumatol 2002 16 761 775 10.1053/berh.2002.0267 12473272

[B2] Burton AK Tillotson KM Main CJ Hollis S Psychosocial predictors of outcome in acute and subchronic low back trouble Spine 1995 20 722 728 10.1097/00007632-199503150-00014 7604349

[B3] Cherkin DC Deyo RA Street JH Barlow W Predicting poor outcomes for back pain seen in primary care using patients' own criteria Spine 1996 21 2900 2907 10.1097/00007632-199612150-00023 9112715

[B4] Shaw WS Pransky G Fitzgerald TE Early prognosis for low back disability: intervention strategies for health care providers Disabil Rehabil 2001 23 815 828 10.1080/09638280110066280 11763278

[B5] Fransen M Woodward M Norton R Coggan C Dawe M Sheridan N Risk factors associated with the transition from acute to chronic occupational back pain Spine 2002 27 92 98 10.1097/00007632-200201010-00022 11805644

[B6] Nykvist F Hurme M Alaranta H Severe sciatica: a 13-year follow-up of 342 patients Eur Spine J 1995 4 335 338 10.1007/BF00300292 8983651

[B7] Andersson GBJ Frymoyer JW, et al The epidemiology of spinal disorders The Adult Spine: Principles and Practice 1997 Philadelphia: Lippincott-Raven 93 150

[B8] Leclaire R Blier F Fortin L Proulx R A cross-sectional study comparing the Oswestry and Roland-Morris functional disability scales in two populations of patients with low back pain of different levels of severity Spine 1997 22 1 68 71 10.1097/00007632-199701010-00011 9122784

[B9] Selim AJ Ren XS Fincke G Deyo RA Rogers W Miller D Linzer M Kazis L The importance of radiating leg pain in assessing health outcomes among patients with low back pain. Results from the Veterans Health Study Spine 1998 23 470 474 10.1097/00007632-199802150-00013 9516703

[B10] Miranda H Viikari-Juntura E Martikainen R Takala EP Riihimaki H Individual factors, occupational loading, and physical exercise as predictors of sciatic pain Spine 2002 27 1102 1109 10.1097/00007632-200205150-00017 12004179

[B11] Tubach F Beaute J Leclerc A Natural history and prognostic indicators of sciatica J Clin Epidemiol 2004 57 174 179 10.1016/S0895-4356(03)00257-9 15125627

[B12] Grotle M Brox JI Veierod MB Clinical course and prognostic factors in acute low back pain: patients consulting primary care for the first time Spine 2005 30 976 982 10.1097/01.brs.0000158972.34102.6f 15834343

[B13] Konstantinou K Dunn KM Sciatica: review of epidemiological studies and prevalence estimates Spine 2008 33 2464 2472 10.1097/BRS.0b013e318183a4a2 18923325

[B14] van Tulder MW Koes BW Bouter LM A cost-of-illness study of back pain in the Netherlands Pain 1995 62 233 240 10.1016/0304-3959(94)00272-G 8545149

[B15] Chiodo A Haig AJ Lumbosacral radiculopathies: conservative approaches to management Phys Med Rehabil Clin N Am 2002 13 609 621 viii 10.1016/S1047-9651(02)00021-9 12380551

[B16] Ashworth J Konstantinou K Dunn KM Prognostic factors in non-surgically treated sciatica: a systematic review BMC Musculoskelet Disord 2011 in press 10.1186/1471-2474-12-208 PMC3287121 21943339

[B17] Hasenbring M Marienfeld G Kuhlendahl D Soyka D Risk factors of chronicity in lumbar disc patients. A prospective investigation of biologic, psychologic, and social predictors of therapy outcome Spine 1994 19 2759 2765 10.1097/00007632-199412150-00004 7899975

[B18] Carragee EJ Psychological screening in the surgical treatment of lumbar disc herniation Clin J Pain 2001 17 215 219 10.1097/00002508-200109000-00005 11587111

[B19] Fritz JM Delitto A Erhard RE Comparison of classification-based physical therapy with therapy based on clinical practice guidelines for patients with acute low back pain. A randomised clinical trial Spine 2003 28 1363 1372 12838091 10.1097/01.BRS.0000067115.61673.FF

[B20] Childs MJD Fritz JM Flynn TW A clinical prediction rule to identify patients with low back pain most likely to benefit from spinal manipulation: a validation study Ann Intern Med 2004 141 920 928 15611489 10.7326/0003-4819-141-12-200412210-00008

[B21] Long A Donelson R Fung T Does it matter which exercise? A randomised control trial of exercise for low back pain Spine 2004 29 23 2593 2602 10.1097/01.brs.0000146464.23007.2a 15564907

[B22] Brennan GP Fritz JM Hunter SJ Identifying subgroups of patients with acute/subacute 'nonspecific' low back pain. Results of a randomised clinical trial Spine 2006 31 6 623 631 10.1097/01.brs.0000202807.72292.a8 16540864

[B23] Hill JC Whitehurst DGT Lewis M Comparison of stratified primary care management for low back pain with current best practice (STarT Back): a randomised controlled trial Lancet 2011 378 1560 1571 10.1016/S0140-6736(11)60937-9 21963002 PMC3208163

[B24] Di Blasi Z Harkness E Edzard E Georgiou A Kleijnen J Influence of context effects on health outcomes: a systematic review Lancet 2001 357 9258 757 762 10.1016/S0140-6736(00)04169-6 11253970

[B25] Stewart MA Effective physician-patient communication and health outcomes: a review Can Med Assoc J 1995 152 9 1423 1433 PMC1337906 7728691

[B26] Konstantinou K Hider SL Vogel S Beardmore R Somerville S Development of an Assessment Schedule for Patients with Low Back-Associated Leg Pain in Primary Care Eur Spine J: A Delphi consensus study 2011 in press 10.1007/s00586-011-2057-2 PMC3389105 22052453

[B27] Hill JC Dunn KM Lewis M Mullis R Main CJ Foster NE A primary care back pain screening tool: identifying patient subgroups for initial treatment Arthritis Rheum 2008 59 5 632 641 10.1002/art.23563 18438893

[B28] Roland MO Waddell G Klaber Moffett J Burton K Main C The Back Book 2002 2 London: The Stationery Office

[B29] Hay EM Dunn K Hill J Lewis M Mason E Konstantinou K A randomised clinical trial of subgrouping and targeted treatment for low back pain compared with best current care. The STarT Back Trial Study Protocol BMC Musculoskelet Disord 2008 9 58 10.1186/1471-2474-9-58 18430242 PMC2377248

[B30] Sowden G Hill J Konstantinou K Subgrouping for targeted treatment in primary care for low back pain: the treatment system and clinical training programmes used in the IMPaCT Back study Family Practice 2011 in press 10.1093/fampra/cmr037 PMC3261797 21708984

[B31] Roland MO Morris RW A study of the natural history of back pain. Part 1: development of a reliable and sensitive measure of disability in low back pain Spine 1983 8 141 144 10.1097/00007632-198303000-00004 6222486

[B32] Patrick DL Deyo RA Atlas SJ Singer DE Chapin AM Keller RB Assessing health-related quality of life in patients with sciatica Spine 1995 20 17 1899 1908 10.1097/00007632-199509000-00011 8560339

[B33] Jordan K Dunn KM Lewis M Croft P A minimal clinically important difference was derived for the Roland-Morris disability questionnaire for low back pain J Clin Epidemiol 2006 59 45 52 10.1016/j.jclinepi.2005.03.018 16360560

[B34] Altman DG Practical Statistics for Medical Research, Volume Section 12.4.10 1991 London: Chapman & Hall Publishers 349

[B35] Dunn KM Jordan K Croft PR Characterising the course of low back pain: a latent class analysis Am J Epidemiol 2006 163 8 754 761 10.1093/aje/kwj100 16495468

[B36] Dunn KM Jordan KP Lloyd M Drangsholt MT Resche LL Trajectories of pain in adolescents: a prospective cohort study Pain 2011 152 66 73 10.1016/j.pain.2010.09.006 20971561 PMC3020286

[B37] Strauss A Corbin J Grounded Theory in Practice 1997 California: Sage

[B38] Dunn KM Jordan KP Croft PR Recall of medication use, self-care activities and pain intensity: a comparison of daily diaries and self-report questionnaires among low back pain patients Prim Health Care Res Dev 2010 11 93 102 10.1017/S1463423609990296

[B39] Grovle L Haugen AJ Keller A Natvig B Brox JI Grotle M The bothersomeness of sciatica: patients' self-report of paresthesia, weakness and leg pain Eur Spine J 2010 19 263 269 10.1007/s00586-009-1042-5 19488793 PMC2899809

[B40] Dunn KM Croft PR The importance of symptom duration in determining prognosis Pain 2006 121 126 132 10.1016/j.pain.2005.12.012 16472916

[B41] Lacey RJ Lewis M Jordan K Jinks C Sim J Interrater reliability of scoring of pain drawings in a self-report health survey Spine 2005 30 16 E455 E458 10.1097/01.brs.0000174274.38485.ee 16103839

[B42] Moss-Morris R Weinman J Petrie KJ Horne R Cameron LD Buick D The Revised Illness Perceptions Questionnaire (IPQ-R) Psychol Health 2002 17 1 1 16 10.1080/08870440290001494

[B43] Nicholas MK The pain self-efficacy questionnaire: taking pain into account Eur J Pain 2007 11 153 163 10.1016/j.ejpain.2005.12.008 16446108

[B44] EuroQol Group EuroQol-a new facility for the measurement of health-related quality of life. The EuroQol group Health Policy 1990 16 199 208 10109801 10.1016/0168-8510(90)90421-9

[B45] Bennett MI Smith BH Torrance N Potter J The S-LANSS score for identifying pain of predominantly neuropathic origin: validation for use in clinical and postal research J Pain 2005 6 149 158 15772908 10.1016/j.jpain.2004.11.007

[B46] Zigmond AS Snaith RP The hospital anxiety and depression scale Acta Psychiatr Scand 1983 67 361 370 10.1111/j.1600-0447.1983.tb09716.x 6880820

