# Supplementary material for: Clinical course, characteristics and prognostic indicators in patients presenting with back and leg pain in primary care. The ATLAS study protocol
Source: BMC Musculoskelet Disord. 2012 Jan 20;13:4. doi: 10.1186/1471-2474-13-4 (PMC3293000; doi:10.1186/1471-2474-13-4)
Supplement: Additional file 1 — Study Flowchart. [file 1471-2474-13-4-S1.DOCX]

**Group (i) intervention/pathway (for patients with mild/improving symptoms):**

The patient will receive brief treatment (one or two sessions) by an experienced physiotherapist with the option to contact the service again if symptoms persist or worsen.

**Group (ii) intervention/pathway (for patients with moderately severe symptoms):**

The patient will receive treatment by a specialist physiotherapist according to individual need. The treatment will include evidence-based physiotherapy techniques and cognitive behavioural approaches. Patients that fail to improve or worsen will be referred to the dedicated spinal service for further assessment and management, including investigations and onward referral to spinal surgeons / pain specialists / rheumatology or other appropriate services/specialty.

**Group (iii) intervention (for patients with severe symptoms):**

Patients in this group will have very severe symptoms and may not be appropriate for physiotherapy interventions and/or cognitive behavioural approaches, or may have not previously responded to physiotherapy interventions. These patients will directly enter the dedicated spinal service for further tests and management. Onward referral to spinal surgeons / pain specialists / rheumatology or other appropriate services/specialty will be arranged as appropriate.

**Follow up with full measures at 4 & 12 months. Monthly follow up with brief measures.**

#### Physio Direct

-Patient from participating GP practice phones Physio Direct

-After usual telephone triage, eligible patients are told about the Community Back and Leg Pain Clinic and informed they will receive an invitation to attend.

**Patient consults GP with low back and leg pain.**

GP enters Read Code on computer & screen prompt appears.

GP has the option to give an information leaflet and tells the patient they will receive a postal invitation to the Community Back and Leg Pain Clinic.

Patient receives invitation and information leaflet.
Telephones to book appointment at Community Back and Leg Pain Clinic.

Appointment confirmation letter and baseline questionnaire sent to patient.

Reminder telephone call 2 days before appointment

Unsuitability flagged on computer and patient not invited to Clinic

### Treatment

Clinic administrator books appointment with appropriate physiotherapist. The treatment pathways will be one of the following:

**Community Back and Leg Pain Clinic**

Clinic administrator greets patient, organises paperwork and introduces nurse.

Nurse informs patient about the study, confirms potential eligibility and gains consent.

Nurse completes brief baseline assessment with consenters to the study.

Patient assessed by study physiotherapist, using pre-agreed standardised assessment schedule. Full eligibility is confirmed and care pathways selected.

GP informed if patient does not book appointment

**Declines participation in study**

Assessment by physiotherapist

GP informed of outcome
