# Supplementary material for: Clinical course, characteristics and prognostic indicators in patients presenting with back and leg pain in primary care. The ATLAS study protocol
Source: BMC Musculoskelet Disord. 2012 Jan 20;13:4. doi: 10.1186/1471-2474-13-4 (PMC3293000; doi:10.1186/1471-2474-13-4)
Supplement: Additional file 2 — Clinical Assessment Form. [file 1471-2474-13-4-S2.DOCX]

Patient sticker

**
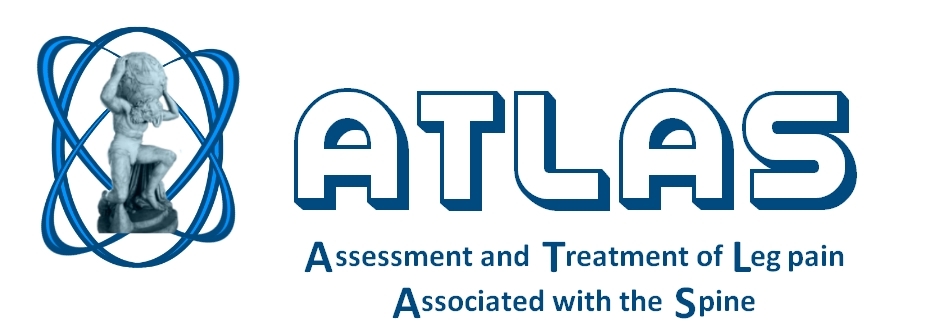
**

**Clinical Assessment Form**

Date of Assessment

Assessor’s Full Name

Assessor’s initials Signature

Patient Eligible for Study: **Yes / No**

**Please state reason if patient non-eligible for study…………………………………**

**…………………………………………………………………………………………..…………………………………………………………………………………………..**

**Clinical History**

Age:

Pain Rating (0 – 10)

LBP: at worst.................

at best...................

average.................

Leg pain:

at worst...............

at best..................

average................

Quality of Pain in Leg

Burning, tingling, sharp, throbbing, toothache, like an electric shock.

Other (please specify)…………….

……………………………………


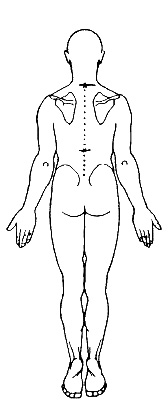


| Presenting condition/symptoms:………………………………………………………………......................................  …………………………………………………………………………………………………………………………..  1.Present since:………………………………………………………………………………………………………….  Reason:…………………………………………………………………………………………………………………..  ……………………………………………………………………………………………………………………………  ……………………………………………………………………………………………………………………………  2. Onset: **Acute / Gradual**  3. Symptoms at onset: **Back / Thigh / Lower Leg**  4. LBP since onset: **Better / Worse / Same** 5. Leg Pain since onset: **Better / Worse / Same**  6. Pins & Needles: **Yes / No** Numbness: **Yes / No**  7. Feeling of weakness in the leg: **Yes / No**  8. Constant symptoms: **Back / Thigh / Lower Leg** 9. Intermittent symptoms: **Back / Thigh / Lower Leg**  10. What is worse: **back / leg** (specify further if necessary)…………………………………………………………...  11. Aggravating Factors: **When Still / Sitting /Standing /Walking / Bending / Sit to Stand / Lying Down /** Other (please specify)……………….......................................................................................................................................... |
| --- |
| 12. Easing Factors: **On the Move/ Sitting / Standing/ Walking/ Bending/ Sit to Stand/ Lying Down/** Other (please specify)...............................................................................................................................................................................  …………………………………………………………………………………………………........................................  13. Functional Limitations: **Yes / No** (what does it stop you from doing – please specify)……………………………  ………………………………………………………………………………………………….......................................  ………………………………………………………………………………………………….......................................  14. Sleep Disturbances: **Yes / No**  Any comments……………………………………………………………………….......................................................  …………………………………………………………………………………………………........................................  15. EMS: **Yes/No**  Any comments……………………………………………………………………….......................................................  16. Unremitting Night Pain: **Yes / No**  Any comments ………………………………………………………………………......................................................  17. BB function: Normal / Other - please comment………………………………….....................................................  ………………………………………………………………………………………………….......................................  18. SA: **No** / **Yes**-please comment ..................................................................................................................................  19. Unexplained weight loss: **Yes / No**  Any comments………………………………………………………………………......................................................  20. General Health: **Good / Fair / Poor**  Any comments……………………………………………………………………….......................................................  21. Any Other Red Flags: **No / Yes** – (please explain).....................................................................................................  …………………………………………………………………………………………………........................................  22. Cough / Sneeze / Strain: **+ve / -ve** (+ve only if it produces patient’s leg symptoms)  23. Gait: steady on feet: **Yes / No**  Any comments……………………………………………………………………….......................................................  24. Previous history of similar LBP: **Yes / No**  Any comments……………………………………………………………………….......................................................  …………………………………………………………………………………………………........................................  25. Previous history of similar Leg Pain: **Yes / No**  Any comments……………………………………………………………………………...............................................  …………………………………………………………………………………………………………………………...  26. Effect of previous treatment for similar symptoms………………………………………………………………….  ……………………………………………………………………………………………………………………………  ……………………………………………………………………………………………………………………………  27. Effect of self-management for similar symptoms……………………………………………………………………  …………………………………………………………………………………………………………………………… |

| 28. Investigations for this problem:  **No investigations / x-Rays** / **MRI** / **Bloods**  Any comments………………………………………………………………………………………………………....  ………………………………………………………………………………………………………………………….  29. Medical History (Past & Present): Chest /Heart / DM /Epil / BP / Ca / steroids / Anticoag / RA / Fract-osteoporosis / serious illnesses / operations……………………………………………………………………  ……………………………………………………………………………………………………………………………  ……………………………………………………………………………………………………………………………  30. Drug History and Effect of Medication on Symptoms:…………………………………………………………….  ………………………………………………………………………………………………………………………….  ………………………………………………………………………………………………………………………….  …………………………………………………………………………………………………………………………..  …………………………………………………………………………………………………………………………..  …………………………………………………………………………………………………………………………..  **Social History**  31.Work: **At work / Off work / Non applicable** (e.g. retired)  (Current details of work, ability to do, effect of symptoms, time off)………………………………………………….  …………………………………………………………………………………………………………………………..  …………………………………………………………………………………………………………………………..  …………………………………………………………………………………………………………………………..  32. Any time off work for previous episodes of back and /or leg pain: **Yes / No**  Any comments………………………………………………………………………………………………………….  …………………………………………………………………………………………………………………………...  33. Benefits: **No / Yes** (please describe)……………………………………………………………………………….  ……………………………………………………………………………………………………………………………  ……………………………………………………………………………………………………………………………  34. Family: (who is at home with them and family situation) (please describe)………………………………………..  ……………………………………………………………………………………………………………………………  ……………………………………………………………………………………………………………………………  ……………………………………………………………………………………………………………………………  35. Physical Activity / Leisure / Sports: (what they do, effect of symptoms on ability to do)………………………….  ……………………………………………………………………………………………………………………………  ……………………………………………………………………………………………………………………………  ……………………………………………………………………………………………………………………………  36. Smoker: **Yes** / **If so, how many a day** **No / Past Smoker**  37. Alcohol Intake: **None / Occasionally / Regular-under recommended limits / above recommended limits** |
| --- |

| **Assessment of Psychological Factors (Yellow Flags)**  38. Evidence of Fear Avoidance: **Yes / No**  …………………………………………………………………………………………………………………………  …………………………………………………………………………………………………………………………  …………………………………………………………………………………………………………………………  39. Evidence of Distress: **Yes / No**  …………………………………………………………………………………………………………………………  …………………………………………………………………………………………………………………………  …………………………………………………………………………………………………………………………  40. Evidence of Low Mood / Depression: **Yes / No**  …………………………………………………………………………………………………………………………  …………………………………………………………………………………………………………………………  …………………………………………………………………………………………………………………………  41. Coping Strategies: **Active / Passive**  ........................................................................................................................................................................................  …………………………………………………………………………………………………………………………  …………………………………………………………………………………………………………………………  42. Work Issues: **Yes / No / Non applicable**  …………………………………………………………………………………………………………………………  …………………………………………………………………………………………………………………………  …………………………………………………………………………………………………………………………  43. Compensation / Litigation: **Yes / No / Non applicable**  ........................................................................................................................................................................................  …………………………………………………………………………………………………………………………  44. Patient’s Future Outlook: **Optimistic / Pessimistic**  …………………………………………………………………………………………………………………………  …………………………………………………………………………………………………………………………  ………………………………………………………………………………………………………………………… |
| --- |

| **Physical Examination**  1.Observation…………………………………………………………………………………………………………..  2. Obvious Abnormalities: **Yes / No**  Any comments…………………………………………………………………………………………………………  …………………………………………………………………………………………………………………………  3. Visible Muscle Wasting: **No / Yes** (if yes please describe)  ……………….…………………………………………………………………………………………………………  4. Gait: **Normal / Antalgic / Unsteady**  Any comments…………………………………………………………………………………………………………  5. Lumbar Shift: **Yes / No**  Any comments…………………………………………………………………………………………………………  **Lumbar Spine Range of Movement**  6. Flexion: normal / limited / hypermobile increase of symptoms: **Yes/No LBP / leg pain**  7. Extension: normal / limited / hypermobile increase of symptoms: **Yes/No LBP / leg pain**  8. Right SF: normal / limited / hypermobile increase of symptoms: **Yes/No LBP / leg pain**  9. Left SF: normal / limited / hypermobile increase of symptoms: **Yes/No LBP / leg pain**  **Neurological Testing; Lower Limbs**     \| **10. Myotomes** \| \| \| \| \| \| \| \| \| \| \| \| \| \| \| \| --- \| --- \| --- \| --- \| --- \| --- \| --- \| --- \| --- \| --- \| --- \| --- \| --- \| --- \| --- \| \|  \| Toe  walking \| \| Heel  walking \| \| Single leg squatting \| \| EHL \| \| Eversion \| \| Inversion \| \| Hip  Flexion \| \| \| R \| L \| R \| L \| R \| L \| R \| L \| R \| L \| R \| L \| R \| L \| \| 0/5 \|  \|  \|  \|  \|  \|  \|  \|  \|  \|  \|  \|  \|  \|  \| \| 1/5 \|  \|  \|  \|  \|  \|  \|  \|  \|  \|  \|  \|  \|  \|  \| \| 2/5 \|  \|  \|  \|  \|  \|  \|  \|  \|  \|  \|  \|  \|  \|  \| \| 3/5 \|  \|  \|  \|  \|  \|  \|  \|  \|  \|  \|  \|  \|  \|  \| \| 4/5 \|  \|  \|  \|  \|  \|  \|  \|  \|  \|  \|  \|  \|  \|  \| \| 5/5 \|  \|  \|  \|  \|  \|  \|  \|  \|  \|  \|  \|  \|  \|  \| \| Comments: \| \| \| \| \| \| \| \| \| \| \| \| \| \| \|      \|  \| Knee jerk \| \| Ankle jerk \| \| \| --- \| --- \| --- \| --- \| --- \| \| R \| L \| R \| L \| \| Normal \|  \|  \|  \|  \| \| Absent \|  \|  \|  \|  \| \| Slightly reduced \|  \|  \|  \|  \| \| Signif. reduced \|  \|  \|  \|  \| \| Brisk \|  \|  \|  \|  \| \| Comments: \| \| \| \| \| | |
| --- | --- | --- | --- | --- | --- | --- | --- | --- | --- | --- | --- | --- | --- | --- | --- | --- | --- | --- | --- | --- | --- | --- | --- | --- | --- | --- | --- | --- | --- | --- | --- | --- | --- | --- | --- | --- | --- | --- | --- | --- | --- | --- | --- | --- | --- | --- | --- | --- | --- | --- | --- | --- | --- | --- | --- | --- | --- | --- | --- | --- | --- | --- | --- | --- | --- | --- | --- | --- | --- | --- | --- | --- | --- | --- | --- | --- | --- | --- | --- | --- | --- | --- | --- | --- | --- | --- | --- | --- | --- | --- | --- | --- | --- | --- | --- | --- | --- | --- | --- | --- | --- | --- | --- | --- | --- | --- | --- | --- | --- | --- | --- | --- | --- | --- | --- | --- | --- | --- | --- | --- | --- | --- | --- | --- | --- | --- | --- | --- | --- | --- | --- | --- | --- | --- | --- | --- | --- | --- | --- | --- | --- | --- | --- | --- | --- | --- | --- | --- | --- | --- | --- | --- | --- | --- | --- | --- | --- | --- | --- | --- | --- | --- | --- | --- | --- | --- | --- | --- | --- | --- | --- | --- | --- | --- | --- | --- | --- | --- | --- | --- | --- | --- | --- | --- | --- | --- | --- | --- | --- |
| Clonus: **No / Yes** (describe)…………………………………………………………………………………………..  Plantars: **downgoing / upgoing / not elicited**  **Right Left**  **12. Sensation** (Pin Prick)  Reduced/absent-describe areas………………………………………………………………………………………..  ………………………………………………………………………………………………………………………….  ………………………………………………………………………………………………………………………….  ………………………………………………………………………………………………………………………….  …………………………………………………………………………………………………………………………  Please tick all  **Normal reduced PP sensation loss of PP sensation total anaesthesia**  relevant boxes:  Allodynia / Hyperalgesia-describe areas………………………………………………………………..…………….  ………………………………………………………………………………………………………………………….  ………………………………………………………………………………………………………………………….  ………………………………………………………………………………………………………………………….  **Right Left**  **13. Neural tension tests**  SLR…………………………………………………………………………………………………………………….  …………………………………………………………………………………………………………………………  Crossover SLR…………………………………………………………………………………………………………  ………………………………………………………………………………………………………………………….  Femoral stretch…………………………………………………………………………………………………………  ………………………………………………………………………………………………………………………….  Slump test……………………………………………………………………………………………………………...  …………………………………………………………………………………………………………………………  **14. Lumbar Spine Palpation Findings** (if present, should be patient’s own pain)  No pain / Local back pain / Radiating pain  Any comments…………………………………………………………………………………………………………  15. Hip Assessment Findings: **Normal / Other** (describe)…………………………………………………………...  ………………………………………………………………………………………………………………………….  16. Any other findings: (please specify)……………………………………………………………………………….  …………………………………………………………………………………………………………………………………………………………………………………………………………………………………………………… | |
| 17. **Clinical Impression** ……………………………………………………………………………………………...  …………………………………………………………………………………………………………………………  …………………………………………………………………………………………………………………………  18. LBP related leg pain: **Yes / No (If No, go to treatment decisions)**  19. LBP with nerve root involvement: **Yes / No**  How confident are you in your clinical impression:  %  (rate on a 0-100% scale, where 100% means absolutely certain/confident):  If you wish to further qualify your rating please use the space below: ….……………………………………………  …………………………………………………………………………………………………………………………  (List **up to 4** most relevant items that led you to your clinical impression/diagnosis)………………………………  …………………………………………………………………………………………………………………………  …………………………………………………………………………………………………………………………  …………………………………………………………………………………………………………………………  …………………………………………………………………………………………………………………………    **Treatment Decisions**  …………………………………………………………………………………………………………………………  ………………………………………………………………………………………………………………………… |  |

**Add any notes/comments you feel necessary**
